# Assessing environmental sustainability of a vital crop in a critical region: Investigating climate change impacts on agriculture using the SWAT model and HWA method

**DOI:** 10.1016/j.heliyon.2024.e25326

**Published:** 2024-02-04

**Authors:** Kazem Javan, Mariam Darestani

**Affiliations:** a School of Civil and Environment Engineering, University of Technology Sydney, Sydney, Australia; b The Department of Civil and Environmental Engineering, University of Western Sydney, Sydney, Australia

**Keywords:** Drought, Climate change impacts, Agriculture, Financial implications, Crop vulnerability, Ardabil plain

## Abstract

Drought is an occurrence that brings about significant changes to the structure of areas. Its influence is especially noticeable in important regions with dry and semi-dry weather patterns, leading to a range of difficulties including interruptions in food distribution systems, lack of water, health problems, economic declines, increased migration, and inadequate energy supply. The Ardabil plain, located in Asia and the northern-western region of Iran, plays a pivotal role in crop productions within an arid environment and holds significant political importance for the country. The main objective of this study is to enhance environmental sustainability in this critical and vulnerable region, particularly in anticipation of imminent droughts. The study focuses on examining the financial impacts on agriculture and selection a crop using the SWAT model, HWA method and climate scenarios under the RCP8.5 pathway for the future period (2040–2050). Results for the near future indicate a notable decline in rainfall of around 38 %, a reduction in wheat production by approximately 25 %, and an increase in temperature of around 30 %. At present, the Ardabil Plain produces a total of 284,182 tons of wheat, with 204,980 tons from irrigated crops and 79,202 tons from rain-fed crops. However, the projected future scenario indicates a decrease in total wheat production to 202,926 tons, with 153,855 tons from irrigated crops and 49,071 tons from rain-fed crops. This decline in production is expected to lead to a total net income loss of approximately -$139,372,437, with -$87,690,344 attributed to irrigated crops and -$51,682,092 to rain-fed crops. The comprehensive hierarchy of crop choices yielded by the HWA method is outlined as follows: barley holds a superior position, followed by wheat, soybeans, and potatoes. The study findings suggest that the availability of water sources in certain regions may prompt a shift in farming land from the north to the south of the plain to promote environmental sustainability.

## 1 Introduction

Drought, a reoccurring occurrence, has substantial consequences for agriculture and various other aspects in Iran, posing a difficult situation that can impede long-term planning and future possibilities [1]. The impact of climate change and increasing temperatures intensify the occurrence of droughts, amplifying both their severity and frequency. This phenomenon is particularly evident in arid and semi-arid regions, where climate change has caused more frequent and severe droughts, leading to diminished water resources for various uses. A study conducted by Ozturk et al. as part of the Coordinated Regional Climate Downscaling Experiment analyzed the influence of climate change on seasonal fluctuations in precipitation and temperature in a section of Asia. The research unveiled a notable warming pattern in surface temperatures in the southeastern Pacific region [2].

The intricate relationship between climate change, escalating temperatures, and drought presents significant challenges, affecting water resources and highlighting the urgency for effective measures to tackle these problems in Iran. In recent times, the country has witnessed a rise in natural disasters, including global warming, climate change, floods, and particularly droughts. Extensive research has been undertaken to examine and understand these occurrences and their implications [1,3]. General Circulation Models (GCMs) are frequently employed to investigate climate change and its consequences. They are utilized to create climatic scenarios for various timeframes, offering valuable insights into the hydrological effects resulting from alterations in climate variables. GCMs play a pivotal role in enhancing our comprehension of how changes in precipitation patterns, temperature fluctuations, and other climate-related factors can impact water resources [[4], [5], [6], [7]]. Mirdashtvan et al. conducted a study on alterations in climatic variables between 2011 and 2040. They employed several Representative Concentration Pathway (RCP) scenarios and evaluated the uncertainty related to downscaling methods in an ecologically sensitive basin situated within Iran's Alborz range [8]. While earlier studies [9,10] have been obtained regarding potential climate change consequences, our comprehension of how climate change affects Iran's hydrological parameters remains restricted. The country has experienced severe events such as rainfall, floods, droughts, and extreme temperatures in recent times [11,12]. The occurrence of these events emphasizes the immediate necessity to further develop our understanding of how climate change is affecting Iran's hydrological conditions.

SWAT is an open-source software that has been developed by the Agricultural Research Service (USDA) since 1990 [13]. It is a semi-distributed model that subdivides the surface of a river basin into various hydrologic response units (HRUs) based on factors such as land use, slope, and soil type [14,15]. Originally designed for large river basins, the SWAT model has found extensive use in various studies focused on water quality, agriculture, and ecology [16]. It is frequently employed to analyze hydrological processes within watersheds, especially in the context of land-use and climate change [13]. However, like other hydrological models, it is subject to a considerable level of uncertainty [17]. The SWAT model has been studied in various locations across Iran. Researchers have utilized this model to assess and understand the hydrological processes, land-use changes, and water resource management in different regions of the country. Shahvari et al., the effects of climate change on water resources were assessed by employing SWAT model under different scenarios of greenhouse gas concentrations, known as Representative Concentration Pathways (RCP 2.6, RCP 6, and RCP 8.5). The research aimed to investigate the potential impacts of different climate change trajectories on water resources and their management [18]. Zakizadeh et al., is employed SWAT model to simulate the impacts of climate change on the hydrological conditions in the Darabad watershed [19]. Emami and Koch focus on assessing the impacts of climate change on the water availability of Zarrine River Basin, which serves as the headwater of Lake Urmia in western Iran. The simulation is conducted using the SWAT hydrological model, and various climate scenarios are considered up to the year 2029, taking into account the presence of the Boukan Dam in the region. Based on the subsequent SWAT simulations for the future period 2012–2029, it is evident that the predicted climate change under all Representative Concentration Pathways (RCPs) will result in a reduction of the inflow to Boukan Dam and the overall water yield of Zarrine River Basin (ZRB) [20].

A decision maker requires a tool to make more effective decisions regarding the outlets of SDMs. Multi-Criteria Decision-Making (MDCM) approaches are deemed effective tools for evaluating water resources management and choosing a comprehensive solution (Diaz-Balteiro, González-Pachón, and Romero 2017; Bell, Hobbs, and Ellis 2003; Wang et al., 2021). The Hybrid Weighted Averaging (HWA) method is one of the MDCM methods (Xu and Da 2003). The preference for HWA stems from its capacity to encompass multiple factors, its adaptability in assigning importance weights, its incorporation of risk attitudes, its enhanced precision in decision-making, and its bolstered transparency. HWA permits decision-makers to thoroughly assess a spectrum of variables, allocate weights according to their significance, and accommodate their risk inclinations. By merging the strengths of diverse weighting methodologies, HWA generates precise and robust decision outcomes. Its transparent approach heightens comprehension and confidence in the decision-making process. HWA furnishes a versatile and all-encompassing framework for decision-making that resonates with the preferences of decision-makers, yielding dependable and transparent results (Mirabi et al., 2014). In developing countries, there is an increasing focus on recognizing and precisely assessing the interconnections and possible conflicts between water resources and agricultural activities (Reddy, Cunha, and Kurian 2018). This acknowledgment holds significant importance when it comes to tackling concerns such as choosing suitable crops for specific watershed areas for particular regions.

Ardabil holds a pivotal position in the country's agricultural sector, being ranked among the leading provinces in agricultural and horticultural output. Its strategic geographic location not only facilitates product exports but also holds political significance within the country's geography. With approximately one-third of the province's population employed in agriculture, it serves as a substantial contributor to the local economy [[21], [22], [23], [24], [25], [26]]. The economic ramifications of climate change-induced disruptions in agriculture can be severe, affecting not only local farmers but also the overall agricultural output and food security of the country. Given the importance of the region's agricultural products for domestic consumption and exports, any disruptions can have significant implications for the national economy. Moreover, Ardabil's strategic position as both an agricultural and tourist destination further adds to its economic significance [27]. The decline in water resources and potential shifts in agricultural productivity may affect Ardabil's position as a major agricultural hub, influencing the country's agricultural exports and trade balance. The Ardabil Plain, in particular, holds great importance within this economic framework. Extensive research has been conducted to address the critical issue of water resources in the agricultural sectors, as they heavily rely on adequate and sustainable water availability [[28], [29], [30], [31], [32], [33]]. However, the region is also affected by drought-related issues and a general trend towards aridification, which poses challenges for agricultural activities [34,35]. This information would be highly valuable for understanding and managing temperature-related factors in farming of the Ardabil plain.

The primary objective of this study is to promote environmental sustainability in a strategically important region that faces the imminent threat of drought. The research focuses on assessing the financial implications on agriculture and selection of a crop in this area and proposes sustainable practices to safeguard the local population's well-being. To achieve this objective, the study employs the SWAT model and climate scenarios specific to Iran's northwest, particularly under the RCP8.5 pathway, covering the period from 2040 to 2050. Through this investigation, the research aims to offer valuable insights and actionable strategies to foster sustainability in the region, ensuring the livelihoods of its residents. By addressing the challenges posed by climate change and implementing appropriate measures, the study strives to contribute to the long-term environmental well-being and economic stability of this critical region.

## 2 Materials and methods

### 2.1 Study area

**Fig. 1:**
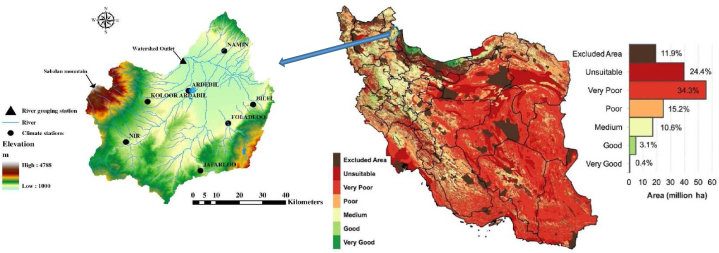
The agricultural viability of Iran's land, as determined by soil and topographic factors (Mesgaran, 2017) (right) and Ardabil Plain (left).

This research centers on the Ardabil plain, located in a semi-arid area in northern Iran. Geographically, Ardabil province holds a strategic position for agricultural product exports, benefiting from its proximity to the Republic of Azerbaijan (Fig. 1). The Sarein station records the highest annual rainfall in the plain, measuring 370 mm, while the Namin station registers the lowest with 220.8 mm and a relative humidity of 63.12 %. With approximately 500,000 inhabitants, the basin relies mainly on agriculture as its primary economic sectors [[36], [37], [38]].

**Fig. 2:**
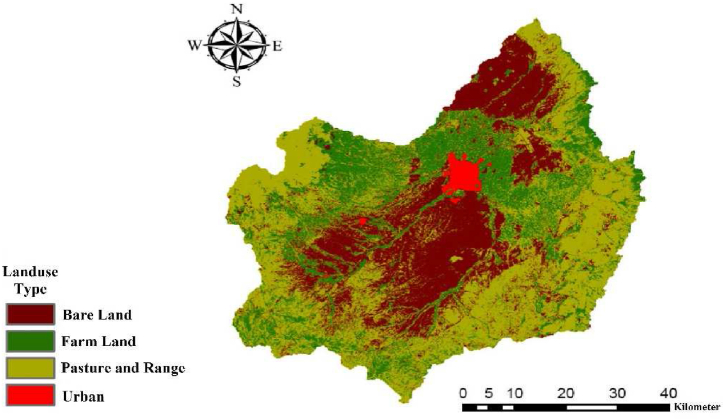
The landuse map of the Ardabil Plain.

The Ardabil plain boasts substantial capabilities for producing a diverse range of agricultural products. The province holds impressive rankings in various agricultural items, being first in lentil production, second in potato production, third in honey, fifth in sugar beet, and seventh in beans, wheat, and barley [37,38]. The province possesses a total of 780,000 ha of registered agricultural lands, which includes 515,000 ha of rain-fed lands, 225,000 ha of non-rain-fed lands, and 40,000 ha specifically allocated for horticultural crops. These agricultural lands collectively yield an impressive output of approximately 4,500,000 tons of diverse agricultural products [37,38] Despite only accounting for slightly over one percent of the country's agricultural land, Ardabil province serves as a crucial center for agricultural production in Iran. Fig. 1 illustrates the agricultural viability of Iran's land, considering soil and topographic factors, highlighting the significance of the Ardabil plain. Within this relatively smaller area, the province contributes more than four percent of the nation's total agricultural output. The agricultural sector in Ardabil currently employs 30 percent of the province's workforce, making it the highest employment sector among development groups. Table 1 presents the farming areas measured in hectares for this study, while Table 2 provides data on the Average Income, Cost, Water Consumption, and Yield per cultivated area for wheat in Ardabil plain, where wheat comprises over 50 percent of the agricultural activities [39]. Ardabil province's exceptional agricultural performance, combined with its advantageous geographical location, underscores its immense potential and significance in the agricultural landscape [37]. The province's abundant natural resources, complemented by the expertise of its farmers, play a crucial role in sustaining its agricultural success and significantly contributing to the overall agricultural productivity of the country [40]. Fig. 2 displays the land use map utilized for the SWAT model in the Ardabil Plain.

**Table 1:** The Farming Areas in Ardabil province and this Study (hectare).

Attributes	Irrigated Farming Land		Rain-fed Farming Land		Total Farming Land		
	Crop	Garden	Crop	Garden	Irrigated	Rain-fed	Total
**Ardabil Province**	185,652	30,780	485,218	370	216,432	485,588	702,020
**Ardabil Plain**	83,156	3492	148,430	39	86,648	148,469	235,117

**Table 2:** Average income, cost and water consumption and yield per cultivated area for wheat per hectare of agriculture in Ardabil plain [39].

Attribute	Income ($)	Cost ($)	Water consumption (m^3^)	Production/Area (Total)	Production/Area (Irrigated)	Production/Area (Rain-fed)
**Amount**	1715.23	709.85	4604.56	1.53	4.25	0.92

### 2.2 Data

**Fig. 3:**
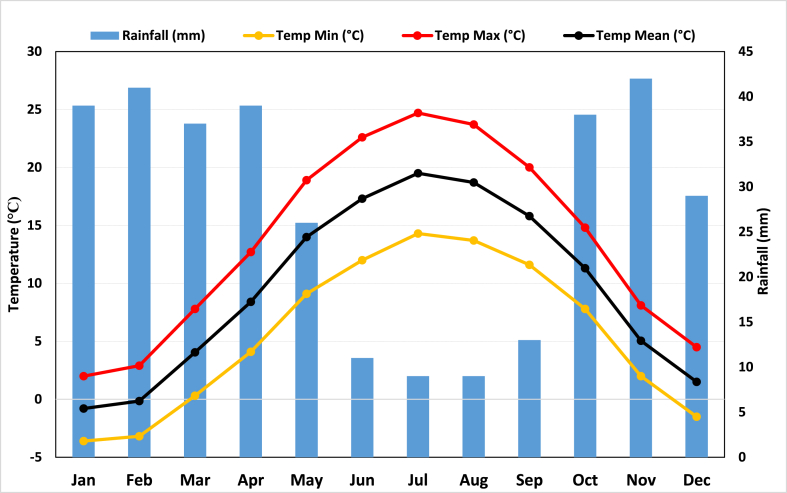
Monthly Temperature and Rainfall in the Ardabil plain.

Temperature and precipitation data were gathered from seven synoptic stations located within the Ardabil plain. The observed climatic data for the period spanning from 2010 to 2020 were acquired from these seven essential synoptic stations in the province. Fig. 3 displays the average monthly temperature and rainfall for all research sites. It is essential to consider that differences in data among various stations can result in systematic errors in rain gauge measurements due to factors like wind, humidity, and evaporation from the gauge's instruments [41,42]. Notwithstanding the endeavors to create an extensive network of measurement stations, there will inevitably be regions where temperature data is not available [43].

For predicting future temperature and precipitation patterns, data from the Coupled Model Intercomparison Project 5 (CMIP5), accessible through the official website of the Intergovernmental Panel on Climate Change (IPCC), were employed. This data resource offers raw data from models, representing past and future climate scenarios across different time periods and conditions. However, one significant limitation of using GCM output is its relatively low resolution, which restricts its applicability for regional studies. To address this, various research institutes have made downscaled versions of GCM output available, providing data on smaller grids to support global access for academic research. For this study, the output from CMIP5 GCMs with a grid size of 0.5° × 0.5° was utilized to assess the near-future period of 2040–2050 [44].

## 3 Research methods

### 3.1 SWAT model

The SWAT model demonstrates the capability to simulate and predict long-term changes in various hydrological elements across large and complex basins under different land use types, soil types, and management practices [45,46]. The smallest unit in this model is the Hydrological Reaction Unit (HRU), derived from a combination of topography, land use, and soil maps [47]. Utilizing ArcGIS software in a shared environment simplifies the operation and usage of this model. The required basic maps for running the model include Digital Elevation Model (DEM) maps, land-use maps, and soil maps, all of which need to be in raster format in GIS [48]. Prior to implementing the SWAT model into the GIS environment, extensive preparation of input data was undertaken. This included gathering and organizing climatic and hydrological data, obtaining topographic maps, compiling soil information, and acquiring land-use data. These preparatory steps were essential to ensure that the model could accurately simulate and analyze the hydrological processes in the study area.

### 3.2 Climate change scenarios and downscaling

Numerous studies have brought attention to the limitations of GCMs because of their coarse resolution and systematic biases [4,49]. The low resolution of these models poses challenges in accurately capturing regional climate dynamics, and the biases can become more pronounced when simulating climate change under global warming conditions. To tackle these issues, bias adjustment is essential for each model [4]. Downscaling is a method utilized to interpolate GCM outcomes in order to fulfill local-scale necessities and diminish biases [50]. This study utilized the bias-correction change factor approach to downscale the 0.5 × 0.5 gridded GCMs to the size of weather stations, resulting in higher resolution and maintaining the seasonal variability of the observed data [51,52]. The study employed Equations 7 and 8, proposed by Yang et al., to assess the agreement between the downscaled data mean and the observed data mean using the change factor approach. These equations were utilized to downscale recorded precipitation and temperature for the projected period of 2040–2050, while considering the historical reference period of 2010–2020 [4].

Equ (1) $${\;P}_{downscaling\;\left(m\right)}=\bar{P_{O_{m}\;}}\times {\left(\frac{\bar{P_{f}}}{\bar{P_{p}}}\right)}_{m}\;m=1,2,\ldots ..,12$$

Equ (2) $$T_{downscaling\;\left(m\right)}=\bar{T_{O_{m}\;}}–\;{\left(\bar{T_{fut}}-\bar{T_{his}}\right)}_{m}m=1,2,\ldots ..,12$$

Regarding the equations and variables employed in the study, the symbol ‘T' stands for temperature, ‘O' represents observational data, ‘f' signifies projected raw data from a climate model for the future period (2040–2050), 'his' represents raw data from a climate model for the baseline period (2010–2020), and ‘m' denotes the months ranging from January to December. The overline notation indicates the mean value [4,44].

### 3.3 Possible frameworks

**Fig. 4:**
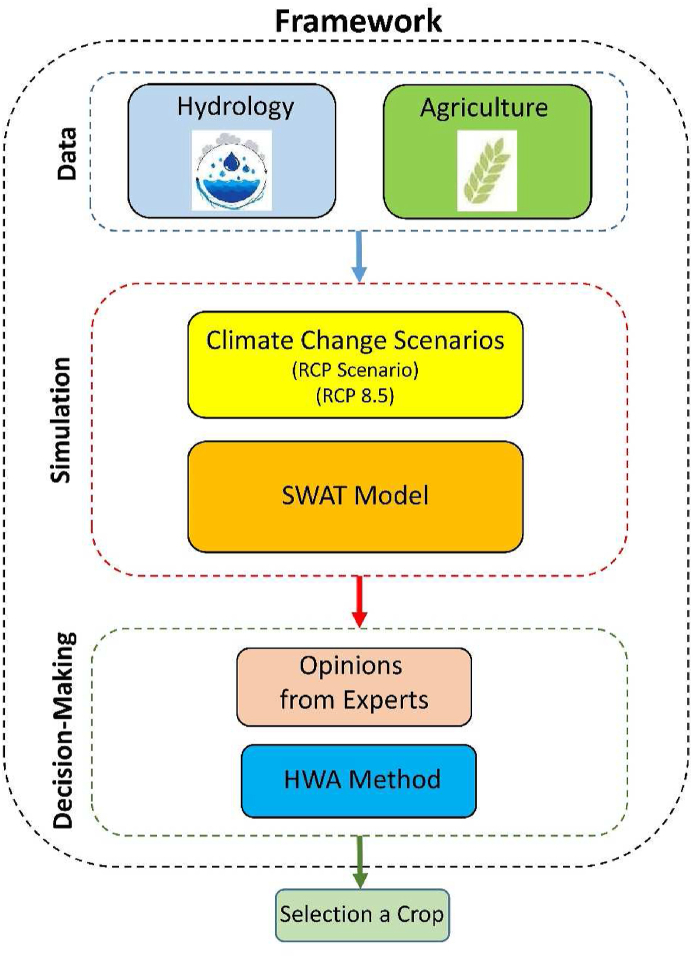
The framework of study.

The proposed research framework focuses on studying the selection a crop in Ardabil plain by utilizing a SWAT model in combination with RCPs and HWA method for decision-makers. The research begins with an introduction to the SWAT mode concept and the importance of considering RCP pathways for understanding climate change impacts. Data collection involves gathering relevant information on water availability, food production, climate stations, and other variables specific to Ardabil plain. The SWAT is calibrated and validated using historical data to ensure its accuracy. Scenario is designed to represent RCP 8.5, and simulations are conducted to analyze the long-term behavior. The HWA method prioritizes crop selection, considering criteria such as water consumption, economic value, environmental effects, and sensitivity. Weights are assigned to each criterion, and the HWA method calculates the weighted average for each crop option under the worst RCP scenario. The SWAT simulations and HWA analysis findings are interpreted, discussing the implications of RCP 8.5 pathway in Ardabil plain. It facilitates informed decision-making for sustainable resource management in the region (Giampietro 2014). The study's framework is illustrated in Fig. 4.

In addition, based on information gleaned from interviews, which showed that reviving just a section of the river discharge and retiring certain geographical regions were seen to be realistic options, the new framework produced for this study. The methods of collecting water for the environment (i.e. acquiring current basin water rights) and restoring only a portion of the Ardebil plain river discharge were studied in these two scenarios. In likely scenarios, the government is projected to acquire new irrigation systems or improve yield technologies for Ardebil plain river discharge restoration in the next 30 years. In one scenario, the Ardebil plain will have a 40 % and 60 % increase in irrigated. For other possible scenarios, land retirement plan will be added in a possible scenario 1. The government may purchase 10 %–20 % of farmed lands for retirement.

## 4 Results

### 4.1 Simulation of hydrological parameters

For validation of SWAT applied various statistical parameters including ME, NRMSE, and R^2^. These parameters were calculated to analyze the outcomes of the techniques by comparing the estimated and measured monthly temperature, precipitation and inflow outlet values. It was observed that the accuracy of the air temperature cross-validations varied from month to month, which could influence the interpolation accuracy for each specific month. The simulation results of the SWAT model revealed favorable performance metrics, with an average R^2^ value of 0.90. Additionally, the ME and NRMSE values for this model were found to be 1.12 and 0.99, respectively. Overall, the R^2^ values for all models indicated satisfactory performance.

**Fig. 5:**
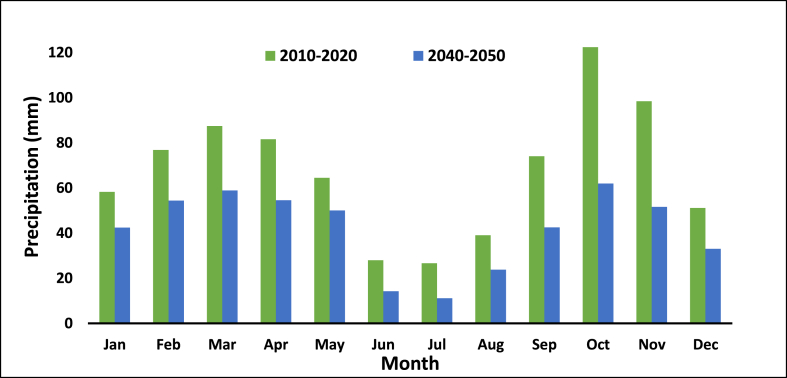
Comparing the average monthly precipitation between a past period and a future period.

In Fig. 5, the average monthly rainfall is compared between the past eleven-year period and the future eleven-year period. The future rainfall generally shows a decrease compared to the base rainfall throughout the year. The highest decrease in rainfall occurs in October, with a future rainfall of 61.99 mm compared to a base rainfall of 122.42 mm, representing a difference of 60.43 mm. The lowest decrease is observed in June, with a future rainfall of 14.24 mm compared to a base rainfall of 27.98 mm, resulting in a difference of 13.74 mm. It is evident that there will be a decline in rainfall by approximately 38 % over the next three decades. The graph highlights that the reduction in rainfall during autumn months is more significant compared to the rest of the year. Additionally, the decrease in rainfall is relatively equal between the winter and summer months.

**Fig. 6:**
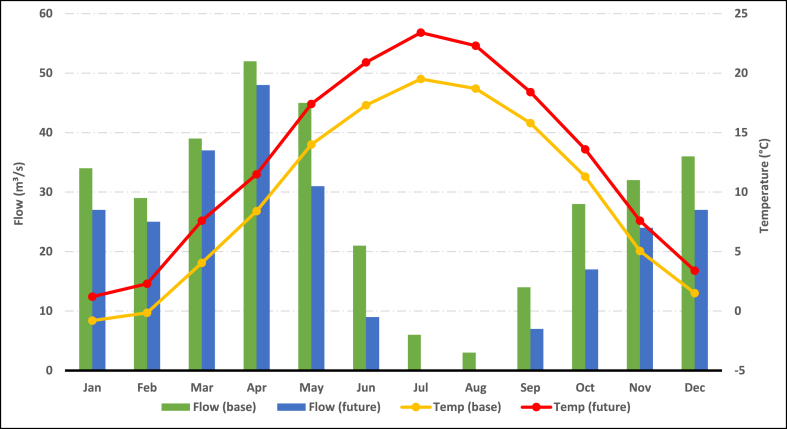
Comparing the average monthly temperature and flow outlet between a past period and a future period.

Fig. 6 shows a compression the average monthly temperature and flow outlet between a past period and a future period. The average base temperature for the year is approximately 9.55 °C, while the average future temperature is around 12.46 °C. This indicates a significant increase of approximately 2.91 °C in the future temperatures compared to the base temperatures with growth 30 %. The highest increase in temperature occurs in July, with a future temperature of 23.4 °C compared to a base temperature of 19.5 °C, representing a difference of 3.9 °C. The lowest increase in temperature occurs in January, with a future temperature of 1.2 °C compared to a base temperature of −0.8 °C, resulting in a difference of 2 °C.

The mean base flow for the year is approximately 28.25 m^3^/s, while the projected future flow is approximately 21 m^3^/s, representing a reduction of approximately 25 %. This signifies a decrease of about 7.25 m^3^/s in the future flow compared to the base flow. The peak flow occurs in April, with a base flow of 52 m^3^/s, and the future flow remains relatively stable at 48 m^3^/s, indicating minimal change. However, the lowest flow occurs in July and August, where both months have no flow in the future scenario, whereas the base flow for July is 6 m^3^/s and for August is 3 m^3^/s. The data reveals a gradual increase in temperatures from January to August, followed by a decrease from September to December. Conversely, water flow experiences a decrease from January to August, reaching its lowest points in July and August, and then begins to recover from September to December. These findings highlight the vulnerability of Ardabil Plain to water scarcity during specific months, which could have significant ramifications for agriculture, ecosystems, and the overall environment.

The data analysis indicates that Ardabil Plain experiences water scarcity during the summer months (July to August) due to reduced flow and lower rainfall. This decline in flow and rainfall could have detrimental impacts on agriculture, ecosystems, and the availability of water resources in the region. In conclusion, the numerical analysis of temperature, flow, and rainfall data in Ardabil Plain demonstrates a warming trend, decreased flow, and reduced rainfall during specific months. The occurrence of water scarcity during the drier months presents significant challenges for the region's water resources and agricultural activities. Implementing sustainable water management practices and closely monitoring the impacts of climate change are essential steps for maintaining a balance between water supply and demand in Ardabil Plain.

**Fig. 7:**
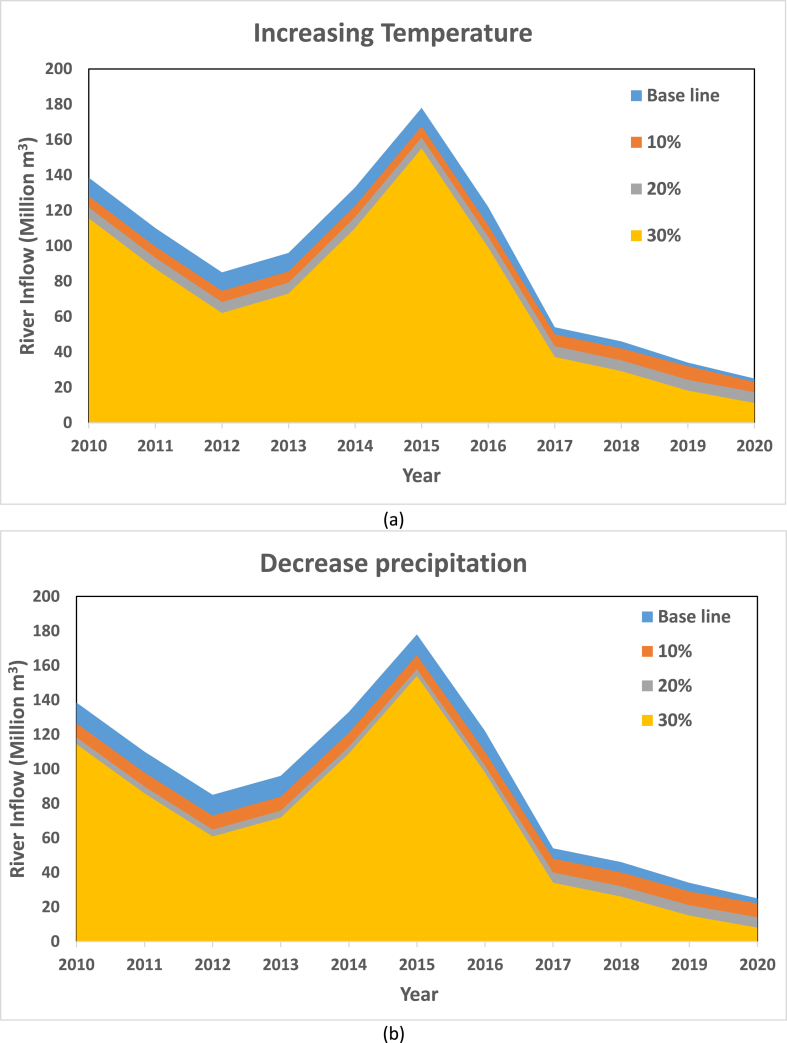
The sensitivity analysis for the various parameters on the Ardebil plain (a) Increasing temperature (b) Decrease precipitation.

The results of the sensitivity analysis for the various parameters on the Ardebil plain from 2010 to 2020 are shown in Fig. 7. It is showed that changes in precipitation have the highest sensitivity. It is obvious that changes in temperature have the lowest. Their contribution to the sensitivity of rainfall estimates is around 0.8 percent of the projected base-case. Furthermore, as compared to temperature variations have a greater impact on River discharge. River inflow is demonstrated to be more responsive to variations in precipitation than temprature from inside the basin.

### 4.2 Agriculture analysis

**Fig. 8:**
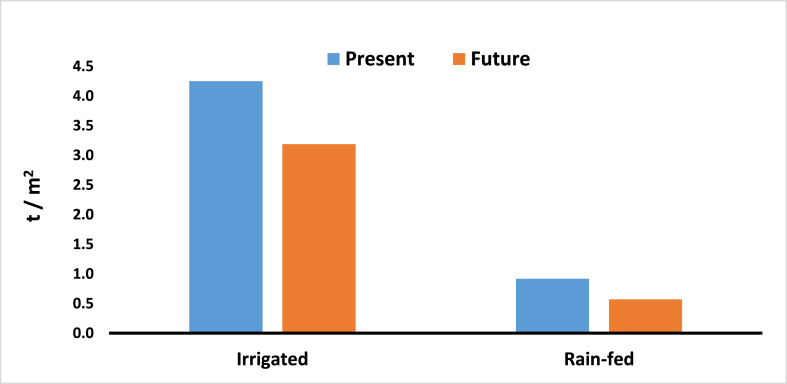
Comparison amount of wheat production to area farming land for two period present and future.

Roughly 58 % of the agricultural land in Ardabil plain is devoted to cultivating wheat, a fundamental staple food for both the people of Iran and the broader region. This section delves into the aspects of production, profitability, and potential losses resulting from climate change on this crucial crop in the area. The analysis assumes that all other factors influencing wheat production will remain constant in the future, except for the amount of rainfall and water utilized for irrigation in wheat cultivation. Fig. 8 illustrates a general declining trend in wheat production volume relative to its cultivation area. Specifically, for irrigated lands, the production value is expected to decrease from 4.25 to 3.19, while for rain-fed lands, it will decline from 0.92 to 0.57 (2040–2050).

**Fig. 9:**
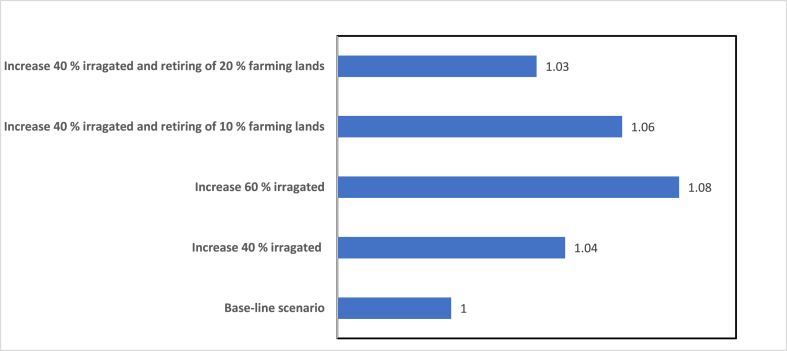
The effect of various scenarios on wheat production.

The impacts of various frameworks on the agricultural economies are also investigated. Fig. 9 illustrates the total wheat output normalized for each scenario. The wheat production data are presented since wheat output is critical in Iran and acts as a primary indication for the regional food business. The total wheat yield in 2019 serves as a reference point against which the results are compared. All of the scenarios indicate an increase in production. With 8 % increases in wheat output, scenario increase 60 % irrigated is the most optimistic. Despite the fact that 10 %–20 % of irrigated agriculture is regarded as retired land, the overall wheat output will improve somewhat by assuming 40 % increase in irrigated and 10 % or 20 % decrease in the farming lands. The scenarios demonstrate that boosting irrigation has a greater influence on wheat production as opposed to abandoning agricultural land. Consequently, efforts towards enhancing irrigation systems can yield more favorable outcomes in terms of setting aside farmland to address water shortages.

Table 3 displays the production volume and alterations in wheat production between the present and future periods. Overall, there is a notable decline in production volume, particularly in a specific region of Ardabil plain. In the present scenario, Ardabil Plain produces a total of 284,182 tons of wheat, with 204,980 tons from irrigated crops and 79,202 tons from rain-fed crops. In the future scenario, the total wheat production is projected to decrease to 209,196 tons, with 153,855 tons from irrigated crops and 49,071 tons from rain-fed crops. Both irrigated and rain-fed wheat productions show a significant decrease in the future. In the case of irrigated crops, there is a reduction of 51,125 tons, and for rain-fed crops, there is a reduction of 30,131 tons. Based on the information provided in Table 3, to calculate the net income from wheat production, you need to multiply the volume of wheat production by the value of $1715.23 US dollars. This will give you the amount of losses incurred due to climate change for wheat production. The data also indicates the projected net income changes resulting from the decrease in wheat production in both present and future scenarios. In future, the total net income will loss for wheat production around -$139,372,437 with -$87,690,345 from irrigated crops and -$51,682,093 from rain-fed crops.

**Table 3:** Amount of Wheat Production in the Ardabil Plain at present period and future period (ton).

Attributes	Present		Future		Changes		
	Irrigated	Rainfed	Irrigated	Rainfed	Irrigated	Rainfed	Total
**Production (ton)**	204,980	79,202	153,855	49,071	−51,125	−30,131	−81,256
**Income ($)**	351,587,056	135,850,072	263,896,712	84,167,979	−87,690,345	−51,682,093	−139,372,437

### 4.3 Decision-making for selection a crop production in the future

The relative significance of each attribute in the decision-making process is indicated by the assigned weights. Particularly within the framework of the SWAT model, focus is directed towards water and power consumption, and the criteria indicators' weights for future considerations have been established. These weights are typically derived from the specific context and priorities set by experts and the SWAT model's framework. By multiplying the attribute values with their respective weights and aggregating the results, a weighted score is generated for each alternative. This procedure facilitates the comparison and evaluation of alternatives based on their performance across various attributes, all while considering their respective levels of importance. The importance of indicators is depicted in Table 4, and the decision matrix can be quantified to ascertain their relative significance.

**Table 4:** Degree of the importance of indicators and the decision matrix.

	Risk Prone		Risk Neutral	Risk Aversion	
	optimistic	fairly optimistic	neutral	fairly pessimistic	pessimistic
**Barley**	**4**	**5**	**5**	**5**	**4**
**Wheat**	**5**	**4**	**4**	**4**	**5**
**Soybeans**	**2**	**2**	**3**	**3**	**3**
**Potatoes**	**3**	**3**	**2**	**2**	**2**

To evaluate how the risk preferences of decision-makers (DMs) influence the selection of the optimal alternative using the HWA method, we conducted a study encompassing three distinct scenarios: optimism (α = 0.1, 0.5), neutrality (α = 1), and pessimism (α = 2, 10). The application of the HWA operator involved the utilization of equation Q(r) = rα, where the risk attitude parameter α is a key factor. With the consideration of a balance coefficient (n = 1), we present the overall values and rankings of alternatives using the HWA operator in Table 3. Under the optimistic risk attitude, both Barley and Wheat receive elevated ratings (4 and 5), indicating their preference when dealing with higher risks. Similarly, under the moderately optimistic risk attitude, Barley and Wheat maintain relatively high ratings (5 and 4), suggesting their suitability in conditions of moderate risk. For the neutral risk attitude, Barley, Wheat, and Soybeans receive intermediate ratings (5, 4, and 3), showcasing their compatibility with neutral risk preferences. Under the moderately pessimistic risk attitude, these same crops receive lower ratings (5, 4, and 3), displaying a preference for less risky alternatives. The pessimistic risk attitude also yields lower ratings for Barley and Wheat (4 and 5), reflecting a preference for safer choices. Across various risk attitudes, Barley and Wheat consistently attain higher ratings, signifying their appropriateness and popularity within the Ardabil Plain, regardless of risk inclinations. In contrast, Soybeans and Potatoes receive lower overall ratings, implying they might be less favored selections for cultivation in the region. Given the anticipated challenges of water scarcity, adverse economic conditions, and escalating environmental effects in the agricultural sector, the optimal choice for cultivation in the Ardabil Plain appears to be Barley. This is reinforced by the rankings depicted in Table 5, which highlight that, overall, the sequence of preferences using the HWA method is barley > wheat > soybeans > potatoes. These empirical findings offer valuable insights into crop selection for agricultural practices in the Ardabil Plain, grounded in considerations of risk attitudes. Barley and wheat emerge as the more favorable options, while soybeans and potatoes are comparatively less favored. These results have the potential to guide decision-making in agricultural endeavors, taking into account both risk preferences and the appropriateness of different crop choices for the specific geographical context.

**Table 5:** Comparison of the final value and rank of each option using the HWA method and different risk levels.

**Attributes**		**Water Consumption**	**Economic value**	**Environmental Effects**
	**Indicator Weight**	**6**	**7**	**5**
**Barley**		**4**	**3**	**8**
**Wheat**		**5**	**5**	**7**
**Soybeans**		**6**	**8**	**4**
**Potatoes**		**7**	**7**	**5**

A range of recommendations is put forth to tackle the challenges within this geographical area. Given the distinctive circumstances of the Ardabil Plain, if the cultivation of wheat and barley persists in the foreseeable future, numerous benefits can be expected. These advantages encompass decreased energy and water consumption, as well as heightened economic profitability. Attaining these benefits hinges on effective planning for alterations in land use and furnishing farmers with essential support and education during this critical phase.

Conversely, if farmers choose to continue with their existing crop preferences, authorities must address the specific conditions and requisites associated with these crops. Primarily, offering sustained financial support to farmers for the development of more efficient irrigation systems like lateral shift, drip, and spray can aid in conservation efforts. However, it is imperative to ensure that these efficiency gains do not lead to the expansion of irrigated agricultural lands, as this could potentially trigger a rebound effect on irrigation efficiency, thereby exacerbating water scarcity. Vigilance in overseeing and managing groundwater extraction is pivotal to prevent excessive usage and the consequent surge in energy consumption. Enhancing pump efficiency has the potential to elevate agricultural profitability, yet it may not inherently conserve water or curtail demand. Alternatively, augmenting irrigation efficiency could serve as a more pragmatic option compared to expanding surface water development in the agricultural sector, provided robust land management protocols are in place to avert new farmland from being irrigated with water resources. The depletion of groundwater storage over the next three decades poses a looming threat to the enduring sustainability and economic viability of irrigated agriculture and food production. Therefore, prudent constraints on the expansion of agricultural land must be thoughtfully enforced. However, the study is subject to limitations due to data paucity, resulting in simplified assumptions within the SWAT. The model excludes significant aspects such as major dam water allocation plans, actual water use and withdrawal in the agricultural sector. Uncertainties surrounding future climate change forecasts, including precipitation, runoff, pan evaporation, and interception coefficient, are addressed by incorporating various RCPs. Notably, the study lacks calibration or testing against the cost of power or water due to the dearth of precise and reliable data. Furthermore, it does not account for unforeseen changes in acreage prompted by fluctuations in crop profitability, the socioeconomic impacts of international sanctions, and their implications for the viability of irrigation modernization.

In summary, the provided information offers a detailed analysis of various factors affecting the Ardabil region, including the impact of climate change, water availability, agricultural productivity, and the tourism industry. The analysis sheds light on potential challenges and consequences that the region may encounter in the near future. Over the next three decades, the Ardabil region is projected to face rising temperatures and changing rainfall patterns. These changes will have significant implications for agriculture, water resources, and tourism. Notably, there is an anticipated 38 % decline in rainfall, particularly during autumn, which could disrupt agricultural activities in the region. The Ardabil plain alone contributes to about 45 % of irrigated crops and 30 % of rain-fed crops in the province, making it vulnerable to serious issues for farmers, especially with the primary crop being wheat. In fact, wheat production is expected to decrease by around 28 %, leading to an estimated total net income loss of approximately -$139,372,437.

## 5 Conclusion

The primary objective of this study is to promote environmental sustainability in a critical region by identifying areas prone to drought in the near future using the SWAT model. The study focuses on the Ardabil basin in 2050 and evaluates the severity of drought. The study's findings reveal significant changes in the future climate of the Ardabil region. Projections indicate a substantial decrease of approximately 38 % in rainfall over the next three decades, affecting both winter and summer months. Average temperatures are also expected to rise, showing a 30 % increase, particularly under the RCP8.5 scenario. A noticeable downward trend in wheat production volume in relation to its cultivation area is evident. Specifically, for irrigated lands, the production value is anticipated to decline from 4.25 to 3.19, and for rain-fed lands, it is expected to drop from 0.92 to 0.57. Overall, there will be a significant reduction in production volume, particularly in a specific region of the Ardabil plain.

Presently, the Ardabil Plain produces a total of 284,182 tons of wheat, with 204,980 tons from irrigated crops and 79,202 tons from rain-fed crops. However, in the future scenario, total wheat production is projected to decrease to 202,926 tons, with 153,855 tons from irrigated crops and 49,071 tons from rain-fed crops. This decline in production will result in a total net income loss of approximately -$139,372,437, with -$87,690,345 attributed to irrigated crops and -$51,682,093 to rain-fed crops. Considering the foreseen obstacles of water scarcity, adverse economic circumstances, and increasing energy consumption within the agricultural domain, the cultivation of barley stands out as the most advantageous option for the Ardabil Plain. According to the HWA approach, the general hierarchy of crop selections is as follows: barley holds the top position, followed by wheat, soybeans and potatoes.

In summary, the Ardabil region is expected to face rising temperatures and shifting rainfall patterns, resulting in significant economic implications. The agricultural sector, crucial for the region and the country, will encounter challenges due to reduced water availability, crop growth, and increased evaporation rates. Farmers may need to relocate their farming areas to more suitable regions, potentially leading to changes in population density. The decline in rainfall and potential shifts in agricultural productivity could affect Ardabil's status as a major agricultural hub, impacting agricultural exports and trade. Proactive measures, including investing in irrigation systems, sustainable farming practices, and water resource management, are essential to adapt to these changing conditions. Long-term planning, policy development, and diversification of the local economy are also crucial to mitigate the economic risks associated with climate change in Ardabil. By exploring alternative sources of income and promoting employment opportunities in other sectors, the region can address the economic challenges posed by climate change effectively. Comprehensive strategies that focus on sustainable agriculture, efficient water management, and economic diversification will play a key role in ensuring the region's long-term prosperity and resilience to climate change impacts.

## Author contributions

Mariam Darestani: Writing – review & editing. Kazem Javan: Writing – original draft, Validation, Software, Resources, Project administration, Methodology, Investigation, Formal analysis, Data curation, Conceptualization

## Data availability

Data will be made available on request.

## Declaration of competing interest

The authors declare that they have no known competing financial interests or personal relationships that could have appeared to influence the work reported in this paper.
